# Profile of Non-communicable Disease Risk Factors Among Young People in Palau

**DOI:** 10.2188/jea.JE20140156

**Published:** 2015-05-05

**Authors:** Chifa Chiang, Singeru Travis Singeo, Hiroshi Yatsuya, Kaori Honjo, Takashi Mita, Edolem Ikerdeu, Renzhe Cui, Yuanying Li, Berry Moon Watson, Gregorio Ngirmang, Hiroyasu Iso, Atsuko Aoyama

**Affiliations:** 1 Department of Public Health and Health Systems, Nagoya University School of Medicine, Nagoya, Japan; 2 Ministry of Health, Republic of Palau, Koror, Palau; 3 Department of Public Health, Fujita Health University School of Medicine, Toyoake, Aichi, Japan; 4 Global Collaboration Center, Osaka University, Suita, Osaka, Japan; 5 Institute for Academic Initiatives, Osaka University, Suita, Osaka, Japan; 6 Public Health Graduate School of Medicine, Osaka University, Suita, Osaka, Japan

**Keywords:** non-communicable disease, WHO STEPS, obesity, tobacco use, Pacific islanders

## Abstract

**Background:**

Although non-communicable diseases (NCDs) have become the predominant health problems of Palauan society, there have been no comprehensive data on NCD risk factors available to develop effective control strategies. Therefore, the first Palauan national STEPwise approach to risk factor Surveillance (STEPS) was completed in mid-2013 to provide information on its adult population aged 25 to 64 years. This study aims to obtain corresponding data from the younger adults aged 18 to 24 years, who remained to be surveyed.

**Methods:**

We conducted an epidemiological study, targeting the 18- to 24-year-old age group. A survey station and a mobile team were established to recruit voluntary participants dwelling in Koror. A slightly modified WHO STEPS instrument was used, including a structured questionnaire for behavioral risk factors, physical measurements, and blood tests.

**Results:**

A total of 356 young people were recruited during the survey. In both sexes, nearly half of the participants were overweight/obese. The prevalence of hypertension was higher in men than in women (17.6% vs 1.7%). Raised blood glucose and impaired fasting glucose were observed in 3.5% and 5.2% of the total participants, respectively. About 36% of the subjects were observed to have raised levels of total cholesterol. More than 70% of the young people were current tobacco users, in terms of all kinds of tobacco products.

**Conclusions:**

The current survey, for the first time, revealed a high prevalence of NCD risk factors, especially overweight/obesity and tobacco use, among young people in Palau. This indicates that swift measures against NCDs are required even in this young age group.

## INTRODUCTION

Over the past decade, the increasing burden of non-communicable diseases (NCDs) in Palau has been recognized as a serious public health threat. As early as the 1970s, notable shifts in dietary patterns and lifestyle changes often associated with NCDs had already been reported.^1^^–^^3^ In 2011, the national mortality data showed that four leading causes contributed to more than two-thirds of all deaths, namely cardiovascular disease (24.3%), cancer (21.4%), chronic respiratory disease (12.7%), and diabetes (9.8%),^4^ indicating that NCDs are the predominant health problems of the islanders.

Several population-based surveys for adult NCD risk factors have been conducted, including the Palau Community Health Assessment (PCHA), which was completed in 2003, and the behavioral risk factor surveillance system (BRFSS), which was initially piloted in 2010, conducted in 2012, and has been adopted as an annual surveillance tool. Although data on various NCD-related behavioral risk factors were collected from these surveys, physical measurements were not included in the BRFSS, and biochemical measurements were not included in either the PCHA or the BRFSS. Accordingly, the Palauan Ministry of Health collaborated with the World Health Organization (WHO) to launch the first comprehensive nationwide survey, the STEPwise approach to risk factor Surveillance (STEPS), in late 2011 and completed the entire data collection in mid-2013.

The WHO STEPS approach has been developed as a simple and standardized method that can be implemented in all countries to monitor NCD risk factors. Using the same standardized questions and protocols to collect small amounts of useful information makes it possible not only to observe within-country trends but also to make comparisons across countries. Its sufficiently flexible framework allows each country to expand on the core modules, and to incorporate optional modules to meet local and regional interests. For low- and middle-income countries, the WHO STEPS survey offers an entry point to begin NCD surveillance activities and helps them develop the capacity of their surveillance systems.^5^^,^^6^

In Palau, the national STEPS survey targeted all adult residents aged 25 to 64 years. Therefore, younger adults aged 18 to 24 years were not its targeted population. This young age group was also not included in various school health surveys conducted in Palau, so this study was carried out to investigate major NCD risk factors among young people aged 18 to 24 years.

## METHODS

The STEPS instrument includes three levels (steps), and within each level, risk factor assessment is divided into core, expanded, and optional items. The Palauan national STEPS covered the core and expanded items of three steps for eight major behavioral and biological risk factors: tobacco use, harmful alcohol consumption, unhealthy diet, physical inactivity, overweight and obesity, raised blood pressure, raised blood glucose, and abnormal blood lipid levels. As described below, we slightly modified the STEPS instrument to fit the characteristics of the young population and other specific interests and needs in Palau for the current study.

### Step 1: questionnaire-based assessment

In addition to basic socio-economic information and all standard modules for self-report behavioral measurements, extra questions were added to assess mental health, sleep habits, and illicit drug use. Moreover, adaptations were made to the standard modules of the STEPS to address specific health priorities and concerns in Palau. For example, findings from the 2003 PCHA showed that over half (58.4%) of Palauan adults were betel nut chewers, and the majority of those individuals (84.3%) chewed betel nuts with tobacco. Questions about betel nut use and use of betel nuts with tobacco were added to the tobacco use module accordingly. The module of dietary behaviors for Palauan national STEPS only included the consumption of fruits, vegetables, and fats and oils. To better understand the nutritional status of the public, we posed questions about the consumption of meat, fish, dairy products, processed/canned foods, and sugar-sweetened beverages.

### Step 2: physical measurements

Step 2 included measurements of weight and height, waist and hip circumferences, and blood pressure. The anthropometric examination was performed without shoes or any heavy clothing. Before measuring blood pressure, participants were asked to sit quietly for about 5 minutes and place their elbows on the table so that the cuff was the level with their hearts. Each participant’s blood pressure measurement was taken three times in the upper arm using automatic digital blood pressure monitors (Omron HEM-7200; Omron Healthcare Co., Ltd., Kyoto, Japan). The three readings of blood pressure were recorded, and the arithmetic mean of the second and third readings was used for the analysis.

### Step 3: biochemical measurements

Capillary whole-blood samples were drawn using the fingertip lancing technique, immediately followed by biochemical tests conducted on portable devices. We used the ACCU-CHEK Aviva blood glucose meter (Roche Diagnostics K.K., Tokyo, Japan) for measuring fasting blood glucose levels and the POCket Lipid (Techno Medica Co., Ltd., Yokohama, Japan) for blood lipid levels. In addition to total cholesterol and fasting triglycerides, HDL-cholesterol was measured and LDL-cholesterol was calculated via the Friedwald Equation.

### Study population and the setting

We referenced data from the Palau Mini-Census 2012 for the study design, because the latest population and housing censuses of Palau, carried out in 2005, might not accurately reflect the current population composition. The national population between 18 and 24 years old was reported as 1681 (793 females and 888 males), and more than 80% of this age group resided in Koror, the most populated urban area in the country. Thus, we defined the study population as adults aged 18 to 24 years living in Koror, and roughly half of the total population within this age group (approximately 600 people) was expected to participate and considered the feasible sample size for our study. We established a survey station at Palau Community College (PCC), located in the center of Koror, to provide optimum geographical access to all potential participants. In addition, PCC is the only institution for college-level education in Palau and the single organization that contains the most members of the target age group (473 students). In order to reach as many potential participants as possible, we also dispatched a mobile survey team to a few local communities and major employers in Koror.

### Staff training

A total of eight staff members of the Ministry of Health joined our study team. Six of them were trained as interviewers for Step 1 and as staff of physical measurements for Step 2. In addition to conducting role-playing interviews between staff members, we recruited a few student volunteers from PCC as interviewees for the questionnaire pretest. Based on feedback from the staff during the training, the questionnaire was revised a number of times. At the end of the training, all of the staff members were confirmed to be able to confidently complete the questionnaire-based interview in English within 35 minutes. For the biochemical measurements (Step 3), the other two members, who have plenty of experience administering blood tests with the Palauan national STEPS, were trained to be in charge of the biochemical station. Because all of the biochemical devices and their reagents were purchased from Japan, we translated the manuals into English for the instruction. These two experienced research team members repeated calibration, sample loading, result reading, and troubleshooting on the devices until they could handle the operations independently.

### Participant recruitment and informed consent

Before the study began, this research project was reported in the local press, *Island Times*, describing the aims and the importance of monitoring the risk factors of NCDs. Promotion and recruitment fliers were distributed to all faculties of PCC and posted to the PCC Newsletter periodical and bulletin boards throughout the campus before and during the survey. Outside the campus, information was accessible through public and private informational boards, government offices in Koror, and all popular online social networking groups. The recruitment lasted for a period of 1 month from the beginning of October 2013. A prepaid cell phone card with a value of ten dollars was given as an incentive for voluntary participation in this survey.

New participants were asked to go to the interview room first (Step 1). Prior to the interviews, adequate explanations of the purpose and the procedures of the study were given from the staff, and signed written consent forms were obtained from each of the entrants. After the face-to-face interviews, participants were directed to the room for physical measurements (Step 2). Following these two steps, all participants were instructed to fast overnight starting at 8 pm and return the next morning to complete biochemical measurements (Step 3). Those who failed to return the next morning for the final step were reminded via their given phone numbers or emails.

### Data entry

Data entry was conducted using standard software, EpiData Entry 3.1 (EpiData Association, Odense, Denmark). A programmed data entry template was developed and pretested by technical staff of the Ministry of Health, and the accuracy of the data entry was verified using a double-entry method.

### Data analysis

We categorized all continuous readings taken from both physical and biochemical measurements according to well-defined standards (see Table 1). Body mass index (BMI) was calculated as weight in kilograms divided by height in meters squared, and then grouped as underweight (BMI <18.5 kg/m^2^), normal weight (BMI 18.5–24.9 kg/m^2^), overweight (BMI 25–29.9 kg/m^2^), and obese (BMI ≥30 kg/m^2^), by applying the WHO criteria. Hypertension was defined as systolic blood pressure ≥140 mm Hg, diastolic blood pressure ≥90 mm Hg, or use of antihypertensive medication, based on the Seventh Report of the Joint National Committee on Prevention, Detection, Evaluation, and Treatment of High Blood Pressure (JNC 7).^7^ Fasting blood glucose levels were classified into three groups based on WHO 2006 criteria, as normal (<110 mg/dL), impaired fasting glucose (IFG; 110–125 mg/dL), and diabetes mellitus (≥126 mg/dL).^8^ Fasting glucose levels were also categorized using American Diabetes Association (ADA) criteria, with a lower cutoff value of 100 mg/dL for normal and 100–125 mg/dL for prediabetes. The classification of blood lipids was performed using the cutoffs described below, presented by the Third Report of the National Cholesterol Education Program Expert Panel on Detection, Education, and Treatment of High Blood Cholesterol in Adults (NCEP ATP III).^9^ Categories for triglyceride levels were normal (<150 mg/dL), borderline-high (150–199 mg/dL), and high (≥200 mg/dL). For total cholesterol levels, categories of desirable (<200 mg/dL), borderline-high (200–239 mg/dL), and high (≥240 mg/dL) were adopted. For HDL-cholesterol levels, low (<40 mg/dL) and high (≥60 mg/dL) levels were defined. Because the portable device employed in our survey had a lower limit of detection of 50 mg/dL for triglycerides, assays below the limit were assigned a value of 50 mg/dL for subsequent analyses. We conducted all data analyses using the statistical software, IBM SPSS Statistics for Windows, Version 21 (IBM Corp, Armonk, NY, USA).

**Table 1.** Body mass index, blood pressure, and blood levels of glucose and lipids among adults aged 18–24 years in Palau, 2013

Factor	Category	Male (*n* = 174)	Female (*n* = 180)	Total (*n* = 354)
		
		*n* (Valid %)	*n* (Valid %)	*n* (Valid %)
Body mass index (kg/m^2^)	<18.5	11 (6.5)	13 (7.3)	24 (6.9)
	18.5–25	79 (46.7)	75 (41.9)	154 (44.3)
	25–29.9	45 (26.6)	50 (27.9)	95 (27.3)
	≥30	34 (20.1)	41 (22.9)	75 (21.6)
	missing	5	1	6

Systolic blood pressure (mm Hg)	<120	30 (17.6)	100 (55.9)	130 (37.2)
	120–129	49 (28.8)	54 (30.2)	103 (29.5)
	130–139	63 (37.1)	23 (12.8)	86 (24.6)
	140–159	27 (15.9)	1 (0.6)	28 (8.0)
	≥160	1 (0.6)	1 (0.6)	2 (0.6)
	missing	4	1	5

Diastolic blood pressure (mm Hg)	<70	67 (39.4)	65 (36.3)	132 (37.8)
	70–79	68 (40.0)	76 (42.5)	144 (41.3)
	80–89	28 (16.5)	35 (19.6)	63 (18.1)
	90–99	6 (3.5)	2 (1.1)	8 (2.3)
	≥100	1 (0.6)	1 (0.6)	2 (0.6)
	missing	4	1	5

Hypertension	no	140 (82.4)	176 (98.3)	316 (90.5)
	yes	30 (17.6)	3 (1.7)	33 (9.5)
	missing	4	1	5

Fasting glucose (mg/dL)	<100	112 (67.9)	136 (76.4)	248 (72.3)
	100–109	36 (21.8)	29 (16.3)	65 (19.0)
	110–125	6 (3.6)	12 (6.7)	18 (5.2)
	≥126	11 (6.7)	1 (0.6)	12 (3.5)
	missing	9	2	11

Triglycerides (mg/dL)	<100	122 (74.4)	136 (77.3)	258 (75.9)
	100–149	30 (18.3)	26 (14.8)	56 (16.5)
	150–199	6 (3.7)	8 (4.5)	14 (4.1)
	≥200	6 (3.7)	6 (3.4)	12 (3.5)
	missing	10	4	14

Total cholesterol (mg/dL)	<160	23 (14.0)	29 (16.5)	52 (15.3)
	160–189	77 (47.0)	88 (50.0)	165 (48.5)
	190–199	31 (18.9)	21 (11.9)	52 (15.3)
	200–239	32 (19.5)	37 (21.0)	69 (20.3)
	≥240	1 (0.6)	1 (0.6)	2 (0.6)
	missing	10	4	14

HDL-cholesterol (mg/dL)	<40	3 (1.8)	1 (0.6)	4 (1.2)
	40–49	10 (6.1)	8 (4.5)	18 (5.3)
	50–59	27 (16.5)	17 (9.7)	44 (12.9)
	≥60	124 (75.6)	150 (85.2)	274 (80.6)
	missing	10	4	14

HDL, high-density lipoprotein.

### Ethical considerations

This study was reviewed and approved by the Bioethics Review Committee of Nagoya University School of Medicine and Institutional Review Board of the Ministry of Health, Republic of Palau. Written informed consent was obtained from all of the participants after adequate explanation of the study.

## RESULTS

A total of 356 adults between 18 and 24 years, with a mean age of 20.2 years, voluntarily participated in the survey. Although all participants completed the questionnaire-based interviews and physical measurements, 13 participants (3.7%) failed to come back for the biochemical measurements. The majority (*n* = 268) of the participants were PCC students, and 46 participants (12.9%) were non-Palauan nationals. Pregnancies were reported from two females, and consequently their biological data were excluded from the analyses for this paper. Across the survey, distinct gender differences in participation were not observed.

Table 1 displays the percentages of biological indicators classified by appropriate criteria. In both sexes, nearly half of the participants were found to be overweight or obese. About one in six male subjects was hypertensive. The prevalence of hypertension was much higher in males than in their female counterparts (17.6% vs 1.7%: *P* < 0.001). Among the normotensive young people, one male reported antihypertensive use during the past two weeks. According to the WHO criteria, 3.5% and 5.2% of the total participants showed fasting blood glucose levels of diabetes mellitus and IFG, respectively; however, prevalence of prediabetes by the ADA criteria was 24.2%. Of the 340 valid subjects, 20.9% had borderline-high or high total cholesterol levels (≥200 mg/dL). Using the WHO recommended classification (≥190 mg/dL), 123 (36.2%) young people had raised levels of total cholesterol. Borderline-high or high levels of triglycerides (≥150 mg/dL) affected 7.6% of participants, and low levels of HDL-cholesterol affected 1.2%.

Approximately 40% of male and 12% of female respondents answered that they currently smoke cigarettes (Table 2). Regarding the use of all kinds of tobacco products, 80% of males and 61% of females were current tobacco users at the time of the survey. A quarter of the participants did not eat at least one serving of fresh fruit and vegetables a day. Only 9.2% of participants ate 5 servings of fresh fruit and vegetables or more per day, which is the WHO recommended lower limit. About 8% of the young population responded that they did not have any vigorous- or moderate-intensity physical activities, including activities at work, traveling to and from places, and recreational activities in their daily life.

**Table 2.** Behavioral risk factors among adults aged 18–24 years in Palau, 2013

Factor	Category	Male (*n* = 174)	Female (*n* = 182)	Total (*n* = 356)
		
		*n* (Valid %)	*n* (Valid %)	*n* (Valid %)
Alcohol drinking	current drinker^a^	116 (66.7)	66 (36.3)	182 (51.1)
	ex-drinker	46 (26.4)	69 (37.9)	115 (32.3)
	non-drinker	12 (6.9)	47 (25.8)	59 (16.6)

Smoking	current smoker	71 (40.8)	22 (12.1)	93 (26.1)
	ex-smoker	63 (36.2)	63 (34.6)	126 (35.4)
	non-smoker	40 (23.0)	97 (53.3)	137 (38.5)

Betel nut and tobacco chewing	current chewer	109 (62.6)	98 (53.8)	207 (58.1)
	non-chewer	65 (37.4)	84 (46.2)	149 (41.9)

Tobacco product use	current user	139 (79.9)	111 (61.0)	250 (70.2)
	non-user	35 (20.1)	71 (39.0)	106 (29.8)

Fruits/Vegetables (servings/day)	<1	34 (20.0)	50 (27.9)	84 (24.1)
	1–2.9	85 (50.0)	93 (52.0)	178 (51.0)
	3–4.9	31 (18.2)	23 (12.8)	54 (15.5)
	≥5	20 (11.8)	13 (7.3)	33 (9.5)
	missing	4	3	7

Physical activity	no	5 (2.9)	25 (13.7)	30 (8.4)
	yes^b^	169 (97.1)	157 (86.3)	326 (91.6)

^a^Those who answered that they consumed an alcoholic drink within the past 30 days or 1 month.

^b^Those who answered that they engage in vigorous- or moderate-intensity physical activities in their daily life.

## DISCUSSION

This is the first comprehensive survey for NCD risk factors, targeting the young age group of 18 to 24 years in Palau. Not only the information on behavioral risk factors collected via questionnaire-based interviews, but also the biological data taken from physical measurements and blood tests, provided the baseline data for the population burdened with NCDs.

Our findings revealed an alarming high prevalence of overweight/obesity in both male and female subjects. The percentage was even higher than the statistics reported from a previous national NCD STEPS carried out in another Micronesian country, the Marshall Islands.^10^ Compared with that survey, in which 23.9% of the age group of 15 to 24 years were overweight and 10.6% were obese, Palau might have double the percentage of obesity (BMI ≥30 kg/m^2^) in young people. Given that Palau and the Marshall Islands are at the same income level, upper-middle income, further studies to investigate the potential related risk factors in lifestyle of these two populations could provide useful clues for NCD prevention and control in this region. Male subjects were more likely to have hypertension than females (17.6% vs 1.7%). Such distinct gender differences have not been reported from other previous surveys, including those conducted in the Marshall Islands (15 to 24 years, 2.2% vs 1.7%) or in the USA (20 to 34 years, 5.8% vs 3.9%).^11^ Interpretation of this result requires further analysis or additional studies on this specific age group. The prevalence of raised blood pressure, raised blood glucose, or abnormal blood lipids was not as high as that of overweight/obesity. This might be due to the young age of the participants; however, as they become middle-aged, the problems connected with these risk factors are expected to gradually begin coming to light. Therefore, the most pressing need for this young age group is to have effective public health interventions addressing body weight control or further obesity prevention.

Among behavioral risk factors for the young people, our findings suggest that tobacco use is the most obvious and serious problem. The proportion of cigarette-smoking participants in the current survey was almost the same as in the Marshall Islands or in Japan,^12^ of which current smokers comprised 22.7% (40.8% of males and 4.5% of females) in the group aged 15 to 24 years and 24.5% (39.2% of males and 12.8% of females) in the group aged 20 to 29 years, respectively. However, taking into account of all types of tobaccos, including smoking and chewing, Palau had an extremely high proportion (70.2%) of tobacco use. Betel nut chewing, a local custom in Palauan society, accounts for the high proportion, because almost all of the young chewers (96.3%) added tobacco to their betel nuts. Thus, to deal with the high rate of tobacco use in Palau, which was rarely observed in other countries of the region, betel nut chewers should be targeted for tobacco cessation interventions. With regard to the other behavioral risk factors, including excessive alcohol drinking, infrequent consumption of fresh fruit and vegetables, and lack of physical activity, subsequent analyses of the association with biological risk factors are required to examine their impacts on the population’s health status.

Young people aged 18 to 24 years have been omitted from most of the surveys in Pacific island countries, despite the fact that many of the NCD risk factors might manifest in early life. By targeting the young age group of adults, our study might provide comparative information for the authorities to combat NCDs in the region. In Palau, there is no continuous health monitoring system, such as a regular health checkup in colleges or workplaces, available for adults. Hence, our survey also made the first attempt to introduce a health checkup system into PCC, the only college-level educational institution in the country. Based on the high turnout (57% of PCC students) in this survey, a regular health checkup system with the WHO STEPS instrument was considered feasible in the college campus. We highly recommend that PCC or the government take the initiative to establish a regular health monitoring system, at least covering behavioral (Step 1) and physical (Step 2) measurements, for their members.

Because of the convenience sampling applied in our survey, a major weakness of the data set was the low participation of non-PCC students (25%). It might be possible that the college students had better access to the information of the survey and more free time to join in than other young people outside the college. This shortcoming suggests that the present findings should be generalized to the greater Palauan population in the 18–24 age group with caution. Although probability sampling was not employed for the current study, these results may still reflect the current status of NCD risk factors and provide valuable information for this specific age group.

In conclusion, the current survey revealed a high prevalence of risk factors for NCDs among young people in Palau. The results indicate that swift measures against NCDs are required even for the young age group of 18- to 24-year-olds, which was not included in the Palauan national STEPS. The findings can serve as baseline epidemiological data and help policymakers in devising proper strategies to reduce the impact of NCDs on the population. Moreover, this first-time comprehensive survey will also offer a reference for the further development of NCD surveillance systems in Palau.
